# The taxonomic identity of *Didymostigma trichanthera* (Gesneriaceae)

**DOI:** 10.3897/phytokeys.157.32577

**Published:** 2020-08-26

**Authors:** Lihua Yang, Chen Feng, Ming Kang, Fang Wen

**Affiliations:** 1 Key Laboratory of Plant Resources Conservation and Sustainable Utilization, South China Botanical Garden, Chinese Academy of Sciences, Guangzhou 510650, China South China Botanical Garden, Chinese Academy of Sciences Guangzhou China; 2 Guangxi Key Laboratory of Plant Conservation and Restoration Ecology in Karst Terrain, Guangxi Institute of Botany, Guangxi Zhuang Autonomous Region and Chinese Academy of Sciences, Guilin 541006, China Guangxi Institute of Botany Guilin China; 3 Gesneriad Conservation Center of China, Guangxi Zhuang Autonomous Region, Guilin 541006, China Gesneriad Conservation Center of China Guilin China

**Keywords:** China, Guangdong, *Didymostigma trichanthera*, *Henckelia anachoreta*, taxonomy

## Abstract

Based on consulting original literature, the examination of specimens, and field investigations, *Didymostigma trichanthera* is shown to be conspecific with *Henckelia anachoreta*. Therefore, *Didymostigma trichanthera* is formally treated as a synonym of *Henckelia anachoreta* here.

## Introduction

The genus *Didymostigma* W.T. Wang (1984) was once considered to be a monotypic genus, having only one species, *D. obtusum* (C.B. Clarke) W.T. Wang (1984). Subsequently, two new taxa, *D. leiophyllum* D. Fang & X.H. Lu (Fang et al. 1994) and *D. trichanthera* C.X. Ye & X.G. Shi (2005) were discovered and described. The type species of this genus, *D. obtusum*, is widely distributed from eastern Guangdong to southern Fujian, China (Wang et al. 1998; Li and Wang 2004; Wei et al. 2010). The other two species, however, are regarded as narrowly endemic species and have only been found at their type localities (Fang et al. 1994; Ye and Shi 2005).

*Didymostigma trichanthera* was simply described based on a single collection (*Chuang-Xing Ye 5960*) from Nankunshan, Guangdong Province, China. Ye and Shi (2005) thought that *D. trichanthera* mainly differs from *D. obtusum* by its lanose fertile anthers, hairy filaments, and the unhidden pistil to the corolla tube. The species status of *D. trichanthera* was once doubted by Wei et al. (2010). They find the calyx of *D. trichanthera* is completely different from the type species *D. obtusum*, but they did not carry out further study on this questionable species. After consulting original literature about this genus, type species, and this so-called new species (Wang 1984; Ye and Shi 2005), carrying out field investigations in Nankunshan, and conducting examinations of type materials of *D. trichanthera*, we are convinced that *D. trichanthera* is not a species belonging to the genus *Didymostigma*. Our detailed morphological comparisons find that this so-called *Didymostigma* species is actually conspecific with *Henckelia anachoreta* (H.F. Hance) D.J. Middleton & Mich. Möller (Weber et al. 2011). Consequently, it is essential to reduce *Didymostigma trichathera* to a synonym of *Henckelia anachoreta*.

## Material and methods

We performed detailed comparisons of type materials between *Didymostigma trichanthera* and the type species of *Didymostigma* (*D. obtusum*), and also between *D. trichanthera* and *Henckelia anachoreta*. The study of specimens was conducted in IBK, IBSC and SYS. We also checked high-resolution digital images of the specimens in A (https://huh.harvard.edu/), BM (http://data.nhm.ac.uk/), E (http://www.rbge.org.uk/), K (https://www.kew.org/), P (https://science.mnhn.fr/institution/mnhn/search), TI (http://umdb.um.u-tokyo.ac.jp/Dshokubu/Tshokubu.htm) and WU (http://herbarium.univie.ac.at/index.htm) by their web service, as well as via online databases, such as the Chinese Virtual Herbarium (http://www.cvh.ac.cn/), JSTOR Global Plants (http://plants.jstor.org/) and Specimens Database of Native Plants in Taiwan (http://www.hast.biodiv.tw/Specimens/specimenQueryC.aspx). Moreover, detailed morphological studies of both *H. anachoreta* and *Didymostigma obtusum* were undertaken based on plants from natural populations at Nankunshan. Some other field observations of these two species were carried out in Guangxi, Guangdong, Fujian of China.

## Results and discussion

**Figure 1.**
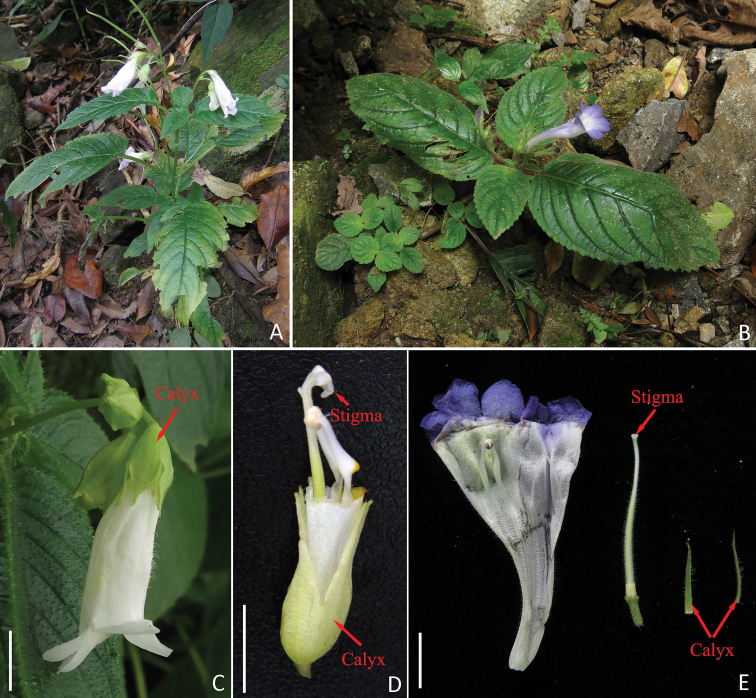
Living plants of *Henckelia anachoreta* (**A, C, D**) and *Didymostigma obtusum* (**B, E**) at Nankunshan. **A, B** habitat **C** side view of flower **D, E** opened corolla showing pistil and stamens. Scale bar: 1 cm. Arrow indicates calyx and stigma.

We did not find any other *Didymostigma* species in Nankunshan in our field work, except for *D. obtusum* (Fig. 1B). However, *Henckelia anachoreta*, a species which is similar to *Didymostigma obtusum* in its vegetative characteristics to some extent, can be easily found here (Fig. 1A). In fact, Nankunshan has been intensively botanized in recent years, because of its high biodiversity (Chen et al. 2017; Xu et al. 2017), and these studies also did not find *D. trichanthera*. As we know, the natural environment of Nankunshan has been well protected since it was listed as a national forestry park in 1993. Nevertheless, *D. trichanthera* has not been found or collected again since it was described in 2005. It is difficult to believe that *D. trichanthera*, if indeed a well characterized species, is represented only by its type collection.

**Figure 2.**
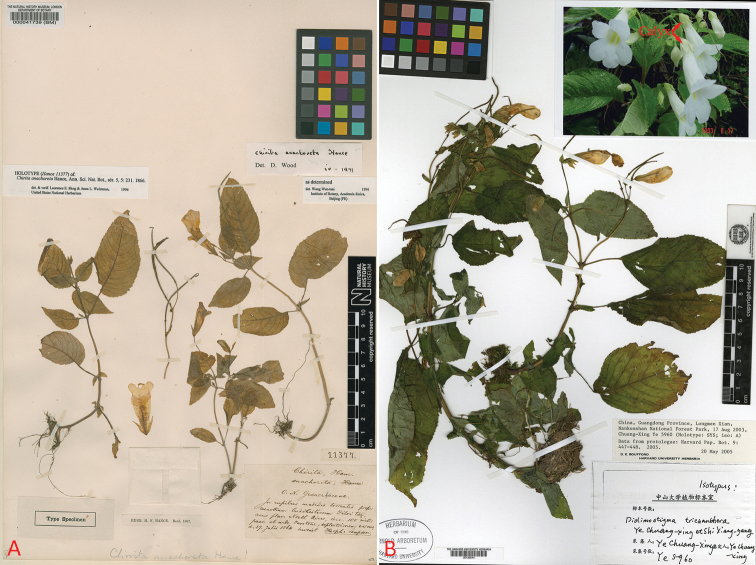
**A** Holotype of *Henckelia anachoreta* (*F.H. Hance 11377*, BM-000041739!) and **B** isotype of *Didymostigma trichanthera* (*Chuang-Xing Ye 5960*, A-00135544!). Arrow indicates calyx.

Unfortunately, the holotype of *D. trichanthera* cannot be found in SYS. According to the isotype (Fig. 2B) preserved in A and the description made by Ye and Shi (2005), we can clearly find that the calyx of *D. trichanthera* is 5-lobed to near middle, with an obvious calyx tube, and the lobes are triangular (Fig. 2B). In contrast, the representative calyx of *Didymostigma* is 5-parted to near base, and the lobes are lanceolate-linear (Fig. 1E; Wang 1984). Additionally, the typical stigma of *Didymostigma* is only ca. 1 mm long (Fig. 1E; Wang 1984). Therefore, although the detailed characteristics of the stigma of *D. trichanthera* have not been clearly observed in the isotype, the description (3–4 mm long) of it in the protologue (Ye and Shi 2005) indicates that it is not a typical character of *Didymostigma*. However, it seems that these two important characters of *D. trichanthera* are well matched with *Henckelia* Spreng. Our detailed comparisons find that there is no obvious difference between the isotype of *Didymostigma trichanthera* (Fig. 2B) and the holotype of *Henckelia anachoreta* (Fig. 2A), and between the images of *Didymostigma trichanthera* given by Ye and Shi (2005) and *Henckelia anachoreta* photo by us at Nankunshan (Fig. 1A, C). In fact, most of the descriptions of *Didymostigma trichanthera* in the protologue, especially the diagnosis characters (lanose fertile anthers, hairy filaments and the unhidden pistil to the corolla tube), are exactly matched with *Henckelia anachoreta*.

All the reasons mentioned above have prompted us to carefully consider that *Didymostigma trichanthera* is conspecific with *Henckelia anachoreta*. As a result, the taxonomic treatment of *Didymostigma trichanthera* needs to be made here.

### Taxonomic treatment

#### Henckelia anachoreta

Taxon classificationPlantaeLamialesGesneriaceae

(H.F. Hance) D.J. Middleton & Mich. Möller in Weber et al. (2011: 774)

4719195C-00C2-543B-ACF3-D93D501EEE94

≡Chirita anachoreta H.F. Hance (1866: 231). Roettlera anachoreta (H.F. Hance) O. Kuntze (1891: 476). Didymocarpus anachoretus (H.F. Hance) H. Lév. (1906: 427).
=Chirita minutiserrulata B. Hayata (1915: 133). Didymocarpus minutiserrulatus (B. Hayata) Y. Yamamoto (1936: 72). Type: China. Taiwan: Boho, July 1911, *Inaba s.n.* (TI, not seen).
=Didymostigma trichathera C.X. Ye & X.G. Shi (2005: 447), syn. nov. Type: China. Guangdong: Longmen County, Nankunshan National Forest Park, 17 August 2003, *Chuang-Xing Ye 5960* (Holotype SYS; Isotype A-00135544!).

##### Type.

China. Guangdong: Qingyuan City, North River, 27 July 1864, *F.H. Hance 11377* (Holotype BM-000041739!; Isotype K-000858355!).

##### Distribution and habitat.

*Henckelia anachoreta* is a common species with a wide distribution in China (Guangdong, Guangxi, Hunan, Taiwan, Xizang, Yunnan), India (Sikkim), Laos, Myanmar, northern Thailand and northern Vietnam (Weber et al. 2011). Plants often grow on moist rocks or ground surfaces in forest or near valley stream sides.

##### Additional specimens examined.

**China.** Guangdong: Haifeng County, 15 August 1935, *W.T. Tsang 25492* (IBSC); Longmen County, 27 October 1981, *G.C. Zhang 280* (HGAS); Maomin County, 2 August 1956, *L. Deng 1751* (HITBC); Qingyuan County, 15 September 1936, *K.Z. Hou 74155* (IBK); Qujiang County, 13 August 1956, *Z. Huang 41855* (IBSC); Ruyuan County, 8 July 2014, *J.M. Li 7840*, *7763* (HEAC); Wengyuan County, 16 August 1933, *X.Q. Liu 2053* (IBSC); Yangshan County, 5 July 1956, *L. Deng 1691* (IBSC). Guangxi: Fangchenggang County, 14 July 1908, *Anonymous s.n.* (PE); ibid. 7 July 1912, *K.K. Chung* (IBSC); ibid. 7 August 1933, *J.L. Zuo 23588* (IBSC); ibid. 25 August 1936, *W.T. Tsang 26748* (IBSC); ibid. 10 September 1936, *W.T. Tsang 26826* (IBSC); ibid. 9 July 2010, *Shiwandashan team 2619*, *2656* (IBK); Gongcheng County, 14 August 1957, *Gongcheng team 195* (IBK); Hengxian County, 15 October 2007, *Y.Q. Su 15915* (GXMG); ibid. 7 September 2008, *Ching-I Peng 21784* (HAST); Jinxiu County, 8 September 1981, *Dayaoshan team 10146* (IBSC); ibid. 19 September 1981, *Dayaoshan team 10317* (IBSC); ibid. 12 September 1981, *Dayaoshan team 10488* (IBK); ibid. 1 November 1981, *Dayaoshan team 12255* (IBK); Jingxi County, 17 September 2010, *Y.S. Huang & L. Wu LYJX0509* (IBK); Lingle County, 15 August 1928, *R.C. Ching 6928* (IBSC); Luocheng County, 15 July 1931, *S.S. Sin 22411* (IBSC); Ningming County, 19 August 2010, *W.B. Xu & W.H. Wu NM396* (IBK); Pingxiang County, 27 August 1986, *Beijing team 0973* (PE); Shanglin County, 6 August 1973, *Y. Wang et al. 67046* (PE); ibid. 19 October 2011, *L. Wu & J.C. Yang D3372* (IBK); Wuming County, 5 August 2010, *L. Wu & R.H. Jiang D0235* (IBK); Xing’an County, 24 September 2014, *Xing’an team 450325140924027LY* (GXMG); Yongfu County, 21 July 1956, *H.F. Qin 700342* (IBK); Zhaoping County, 11 August 1957, *C.Z. Jiang & M.S. Xia 4069* (IBK). Hunan: Guidong County, 19 September 1977, *B.G. Li 5533* (IBSC); Jiangyong County, 8 July 1959, *P.X. Tan 62211* (IBK); 12 July 1959, *P.X. Tan 63671* (FJSI). Taiwan: Kaohsiung hsien, 19 September 1991, *C.C. Wang 588* (HAST); ibid. 13 September 1997, *W.L. Chiou and K.C. Yang s.n.* (WU); ibid. 16 September 2000, *C.I. Peng 18073* (HAST); ibid. 12 August 2008, *C.I. Huang 3463* (HAST); ibid. 6 November 1991, *C.I. Peng 14739, 14770* (HAST); Pingtung hsien, 20 September 1990, *W.P. Leu 551* (HAST); ibid. 23 August 2006, *C.I. Huang 2831* (HAST); ibid. 8 October 2011, *P.F. Lu 22985* (HAST). Xizang: Jilong County, 14 September 2008, *L.M. Gao et al. GLM-081579* (KUN). Yunnan: Cangyuan County, 27 August 2013, *J.M. Li 9485* (HEAC); Hekou County, 27 November 1992, *Y.Z. Wang 92065* (PE); ibid. 18 August 1993, *Y.M. Shui 003411* (PE); ibid. 2 October 2003, *J.M. Li 1022* (PE); ibid. 9 October 2011, M.T. Liu *LMT2011025* (PE); ibid. 17 August 2013, *Z.J. Qiu et al. QZJ-0957* (PE); Jinping County, 12 August 1951, *P.Y. Mao 314* (PE); ibid. 21 September 2006, *L.M. Gao GLM-06283, GLM-06287* (KUN); ibid. 8 September 2012, *Jinping team 5325300650* (IMDY); Luchun County, 30 September 1973, *D.D. Tao 635* (KUN); ibid. 18 October 2000, *Y.M. Shui & W.H. Chen 13123* (KUN); ibid. 23 October 2000, *Y.M. Shui & W.H. Chen 13747* (KUN); ibid. 25 October 2000, *Y.M. Shui & W.H. Chen 13905* (KUN); Maguan County, 19 August 2013, *Z.J. Qiu et al. QZJ-0962* (PE); ibid. 18 September 2013, *P.W. Li LPW2013144, LPW2013143* (PE); Malipo County, 10 August 2004, *J.M. Li LJM-2004-54* (PE); ibid. 28 August 2012, *P.W. Li LPW2012016* (PE); Menghai County, 24 August 2011, *J.M. Li 82412* (HEAC); Menglian County, 6 August 1973, *Menglian team 9967* (KUN); ibid. 14 August 1973, *Menglian Team 10172* (KUN); Mengla County, 23 October 1959, *X.W. Li 13540* (KUN); Pingbian County, 9 July 1934, *H.T. Tsai 62481* (PE); ibid. 18 September 1939, *Q.W. Wang 81896* (KUN); ibid. 20 September 1939, *Q.W. Wang 81981* (KUN, PE); ibid. 28 September 1954, *K.M. Feng 4697* (PE); ibid. 18 September 2012, *Pingbian team* (IMDY); Wenshan County, 14 August 1947 *K.M. Feng 11242* (PE, IBSC); ibid. 20 August 1947, *K.M. Feng 11376* (PE); Xichou County, 29 August 1947, *K.M. Feng 11450* (PE, IBSC); Yanshan County, 19 October 1939, *Q.W. Wang 84483* (PE). **Vietnam.** Hà Tây: Mont-Bavi, 22 July 1886, *Anonymous s.n.* (P); ibid. 4 September 1886, *Anonymous s.n.* (P). Ha Giang: Vi Xuyen Dist., 7 September 2000, *Harder, D.K., Hieu, N.Q., Du, N.V. 5302* (E). Thanh Hoa: Ba Thuoc Dist., 9 October 2003, *Averyanov, L.; Loc, P.K.; Doan, D.T.; Vinh, N.T. HAL4197* (E). Tonkin: Sai Wong Mo Shan, 18 July-9 Sepetember 1940, *W.T. Tsang 30389* (E). **Laos**. Khammouan: Kaeng Meaung landing on Nakai Nam Theun, 21 October 2005, *Newman, M F; Thomas, P I; Armstrong, K E; Sengdala, Khamphone & Lamxay, Vichith LAO 385* (E). **Myanmar.** Haungrys: 15 August 1919, *Kingdon-Ward, F. 3536* (E). **Thailand.** Nakhon Ratchasima: Khao Yai Nat. Park, 22 October 1969, *C.F. van Beusekom, C. Charoenpol 1833* (P); Siam: September 1910, *Q.J.G. Kew 1417* (P).

## Supplementary Material

XML Treatment for Henckelia anachoreta
